# Temporal coordination in joint music performance: effects of endogenous rhythms and auditory feedback

**DOI:** 10.1007/s00221-014-4140-5

**Published:** 2014-11-16

**Authors:** Anna Zamm, Peter Q. Pfordresher, Caroline Palmer

**Affiliations:** 1 Department of Psychology, McGill University, 1205 Dr. Penfield Avenue, Montreal, QC H3A 1B1 Canada; 2 Department of Psychology, University at Buffalo, State University of New York, Park Hall 337, Buffalo, NY 14260 USA

**Keywords:** Joint action, Temporal coordination, Endogenous rhythms, Auditory feedback, Sensorimotor integration

## Abstract

Many behaviors require that individuals coordinate the timing of their actions with others. The current study investigated the role of two factors in temporal coordination of joint music performance: differences in partners’ spontaneous (uncued) rate and auditory feedback generated by oneself and one’s partner. Pianists performed melodies independently (in a Solo condition), and with a partner (in a duet condition), either at the same time as a partner (Unison), or at a temporal offset (Round), such that pianists heard their partner produce a serially shifted copy of their own sequence. Access to self-produced auditory information during duet performance was manipulated as well: Performers heard either full auditory feedback (Full), or only feedback from their partner (Other). Larger differences in partners’ spontaneous rates of Solo performances were associated with larger asynchronies (less effective synchronization) during duet performance. Auditory feedback also influenced temporal coordination of duet performance: Pianists were more coordinated (smaller tone onset asynchronies and more mutual adaptation) during duet performances when self-generated auditory feedback aligned with partner-generated feedback (Unison) than when it did not (Round). Removal of self-feedback disrupted coordination (larger tone onset asynchronies) during Round performances only. Together, findings suggest that differences in partners’ spontaneous rates of Solo performances, as well as differences in self- and partner-generated auditory feedback, influence temporal coordination of joint sensorimotor behaviors.

## Introduction

Many common behaviors, from speaking to walking, require that individuals use sensory information to plan and guide movement. Successful sensorimotor integration occurs when individuals use sensory information to guide motor commands, and adjust their movements when feedback does not match intended sensory outcomes. Sensory-motor links are critical to motor control: Deficits in sensory processing are associated with disorders of motor control (Brown et al. 2005; Laszlo and Bairstow 1983; Stenneken et al. 2006). Sensorimotor integration is a particularly complex task during the production of auditory-motor behaviors such as speech and music, in which integration of sensory feedback with motor commands must occur quickly. When auditory feedback is delayed by a fraction of a second during production of speech and music, the timing of actions is disrupted (Finney 1997; Jones and Striemer 2007; Pfordresher and Palmer 2002).

Although most work on auditory-motor integration focuses on individual performance, there are many auditory-motor behaviors in which individuals must coordinate their actions with others, such as conversational speech and ensemble music performance. Sensorimotor integration between individuals poses more challenges than integration within individuals; performers must integrate sensory and motor information from themselves and from a partner who often generates different auditory feedback from one’s own. Ensemble music performance is a prime example: Musicians must produce tone sequences while integrating auditory information from themselves and a partner to achieve synchronous timing. Furthermore, musicians often differ in their spontaneous rates of performance for the same musical piece, which can influence the ease with which they synchronize with their partner (Loehr and Palmer 2011). We investigate how two factors influence musicians’ temporal synchronization during duet piano performance: individual differences in spontaneous performance rates and the auditory feedback received from oneself and one’s partner.

Several studies suggest that temporal coordination between individuals in joint tasks is influenced by individual differences in endogenous (internal) timing mechanisms. In biology, endogenous rhythms refer to periodic behaviors or processes that occur in the absence of change in external stimulus conditions (Bunning 1956). Endogenous rhythms are thought to influence individual differences in temporal processes across a range of behaviors, including walking rates (Murray et al. 1964), applause (Néda et al. 2000), and spontaneous finger-tapping (Fraisse 1982; McAuley et al. 2006; Moelants 2002), and have been associated with synchronization abilities in music performance (Loehr and Palmer 2011). Individual differences in musicians’ synchronization abilities have been modeled by the amount of coupling between endogenous rhythms and external stimuli (Loehr et al. 2011). Furthermore, individuals with similar spontaneous tempi in pendulum swinging tasks tend to synchronize their movement rates with a partner (Lopresti-Goodman et al. 2008; Richardson et al. 2005), and speakers’ entrainment to other speakers’ rates is modulated by their spontaneous speech rates (Jungers et al. 2002). Some evidence suggests that endogenous rhythms are instantiated in neural oscillations that are modulated by temporal contexts (Henry and Hermann 2014) and that entrain to auditory rhythms present in music and speech (Nozaradan et al. 2011, 2012; Tierney and Kraus 2013). Temporal coordination of ensemble music performance may also be constrained by differences in performers’ endogenous rhythms (spontaneous rates of Solo performance): We investigate whether individual differences in spontaneous rates of Solo performance influence synchronization of actions with a partner during ensemble music performance.

Another factor that may play a critical role in temporal coordination of ensemble music performance is the auditory feedback generated by oneself and by one’s partner. Studies of duet piano performance suggest that auditory feedback from a partner is important to successful coordination: Removing auditory feedback about a partner’s performance leads to reduced synchronization and adaptation (Goebl and Palmer 2009). Temporal coordination changes in different ways when self-feedback is removed: Individuals adapt more to their partner’s timing in duet performance when they cannot hear themselves, than when they can (Goebl and Palmer 2009); similar findings were reported in dyadic finger-tapping tasks (Konvalinka et al. 2010). Together, these findings suggest that feedback from a partner may be more critical than self-feedback when coordinating actions with a partner. To test the importance of self-feedback to joint performance, we compare temporal coordination during duet performances when performers hear full auditory feedback from themselves and their partner (Full) or only feedback from their partner (Other). Temporal coordination should be reduced in the absence of self-feedback, if performers rely on self-feedback during joint performance. If coordination depends only on feedback from one’s partner, then performance should be unaffected by the removal of self-feedback. We also investigate whether self-feedback is more or less important when one is the Leader or Follower: Duet pianists were assigned to the role of Leader or Follower, and Leaders were responsible for maintaining the musical tempo.

**Fig. 1:**
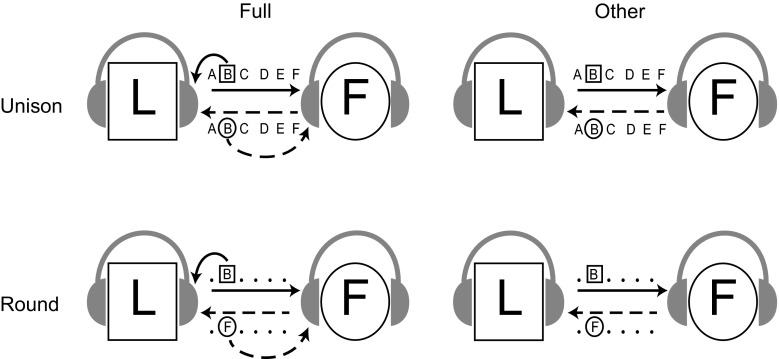
Schematic depicting Feedback and Performance conditions during duet performance. *Squares* represent Leaders, *circles* represent Followers. The *letter series* represent musical sequences; *squared* and *circled* *letters* indicate currently produced tones by Leader and Follower, respectively. During Unison performances, Leader and Follower perform the same tone sequence at the same time. During Round performances, Leaders and Followers perform the same tone sequence at a temporal offset of four tones. *Solid* and *dashed arrows* represent auditory feedback associated with Leader’s and Follower’s keystrokes, respectively. *Curved* and *straight arrows* represent self- and partner-produced sources of auditory signals, respectively

Temporal coordination in joint music performance may also be influenced by the relationship between the contents of auditory feedback produced by the two performers. Ensemble musicians frequently produce distinct but related pitch sequences, such that the content of partner-generated feedback differs from, but is related to, self-generated feedback. Research suggests that performance is disrupted (errors of sequencing and timing) when musicians hear auditory feedback that is related to tones they intend to produce (Pfordresher 2005; Pfordresher and Palmer 2006). Specifically, disruption is large when performers hear auditory feedback that is *serially shifted* relative to their own actions, such that feedback tones correspond to past or future events in their musical sequence (Pfordresher and Palmer 2006). This disruption has been taken as evidence for similarity-based interference arising from conflict between intended and heard events. Similarity-based interference can arise naturally in ensemble music performance when auditory feedback corresponds to past or future events in one’s own productions. A naturalistic version of serially shifted feedback occurs when performers play musical canons or rounds, as shown in Fig. 1: Each performer produces the same tone sequence at a constant temporal offset, so that feedback from a partner is a serially shifted copy of one’s own musical sequence. The temporal lag between the two performers’ sequences further emphasizes the ‘Leader’ and ‘Follower’ roles, as the performer who begins is the Leader and is responsible for maintaining the rate of the musical performance. We compared duet performances of musical Rounds with performances in which partners produce the same tone sequence at the same time (Unison). If hearing past or future events relative to one’s own musical sequence leads to similarity-based memory interference, then temporal coordination during Round performances should be reduced by comparison to Unison performances.

The current study investigated how differences in partners’ spontaneous performance rates and auditory feedback influenced synchronization of joint performance. Pianists performed melodies independent of a partner (Solo), at the same time as a partner (Unison), or at a temporal offset (Round). The alignment of self-produced and partner-produced auditory information was manipulated across Unison and Round conditions, while the auditory and motor information associated with one’s own actions was held constant (see Fig. 1). Access to self-produced auditory information was also manipulated: Performers heard either feedback from both themselves and their partner (Full), or only feedback from their partner (Other) in duet performances. Performers’ Solo performance timing was compared with duet synchronization, to assess the role of spontaneous production rates on synchronization. Duet pianists with similar spontaneous rates in Solo performance were expected to show greater temporal coordination during duet performances than those with dissimilar spontaneous rates. Unison performances were predicted to yield better temporal coordination between performers relative to Round performance, due to similarity-based interference from the Round manipulation. Finally, temporal coordination was expected to be reduced during Other feedback conditions compared with Full feedback conditions, if performers rely on self-feedback during joint performance.

## Methods

### Participants

Thirty-two pianists (mean age 24, range 18–37 years, 16 male) with at least 6 years of private piano instruction (*M* = 12, range 7–20 years) were recruited from the Montreal community. Participants were randomly paired and had no prior knowledge of each other. Four participants reported having absolute pitch, and four reported that they were left-handed. No participants reported any history of hearing impairment. All participants gave informed consent according to procedures approved by the Institutional Review Board of McGill University. Pianists completed a memory test in which they first performed a short melody from notation accurately and then from memory without error, in order to participate.

### Stimulus materials

Two melodies designed to be performed as a musical round were employed: The first melody, adapted from an isochronous Western European song composed by Thomas Tallis (Piece 1), contained eight bars of binary 4/4 meter (32 quarter notes), with four quarter note tones between the entrance of the first performer’s part and the second performer’s part. The second melody, adapted from a primarily isochronous Western European folk song “Lachend” by an anonymous German composer (Piece 2), also contained eight bars of binary 4/4 meter (32 quarter notes), with four quarter note tones between the entrances of the two pianists’ parts. Suggested notated fingerings were indicated in the musical notation, based on recommendations of four skilled pianists, in order to control for possible differences in motor movements.

### Equipment

The pianists performed on two Roland RD-700 keyboards (Roland Corporation, Los Angeles, CA, USA) positioned about three feet apart to face each other. Piano keystrokes were recorded with Cubase software (Steinberg Media Technologies 2010), which received MIDI input from both pianos via an Edirol Studio Canvas SD-80. The audio data from each keyboard were sent to a mixer (Mackie1604 VLZ) and sounded with a St Concert I timbre on participants’ headphones (AKG K-271) via a headphone amplifier (Behringer Powerplay Pro 8). Metronome pulses were generated in Cubase with a percussion timbre (GM Percussion) that sounded at 500 ms inter-onset intervals (IOIs) over participants’ headphones. All experimental sessions were video-recorded with a JVC Everio camcorder.

### Design

The experimental design was based on a Performance (Unison or Round) × Feedback (Full or Other) × Role (Leader/Follower) × Trial (3) repeated measures design, with an additional Solo condition for assessing performance timing independent of a partner. The musical piece (Piece 1/Piece 2) was assigned to co-vary with the Role factor for each participant (see “Procedure”). Each pianist performed the Solo performances first, followed by the Unison duet conditions, and then the Round duet conditions. In Unison and Round conditions, pianists were assigned randomly to the role of ‘Leader’ or ‘Follower’ such that Leaders began performing and Followers began four tones later in Round performances, as shown in Fig. 1. Auditory feedback was manipulated in two conditions: Each performer heard full auditory feedback (Full) or only feedback from their partner (Other). All pianists performed the four Duet conditions in the following order: Unison-Full, Unison-Other, Round-Full, Round-Other, in order to optimize performance in the most difficult conditions. Three trials were completed in each condition. Following the four Duet conditions with the first stimulus melody, the two pianists switched Leader/Follower roles and repeated the four Duet conditions with the other stimulus melody. The order in which pianists performed stimulus melodies was counterbalanced across duet pairs.

### Procedure

Pianists were sent the stimulus melodies prior to the experiment, with the instruction to practice until the melodies were memorized. When pianists arrived at the laboratory, they were seated at two pianos and could not see their partner’s hands. They wore closed headphones through which they could hear only their own feedback and could not hear their partners’ keystrokes. After 5 min of rehearsal with the notated score present, one pianist left the room to complete a musical background questionnaire while his or her partner completed a memory test in which they had to perform the rehearsed melody in the absence of a notated score. If pianists were unable to perform without errors, they were allowed to practice again for up to 5 min and repeat the test. Only pianists who performed without pitch errors by the second performance completed the study. Pianists then performed three Solo trials in which they were instructed to perform the melody at a comfortable rate, four times in succession without stopping. Once the first pianist completed the memory test and three Solo performance trials, the other member of the duet pair completed the memory test and Solo performance trials.

In the duet performance conditions, the pianist seated at one keyboard (left side) was assigned the role of ‘Leader’, and the other pianist the role of ‘Follower’. The Leader was told that they should set the pace of each performance within the constraints of the initial four-beat (500 ms) metronome cue that sounded before each duet trial, and the Follower was told to follow the pace maintained by the Leader. Pianists then completed four duet performance conditions with the first melody. The pianists then repeated the entire procedure for the second melody, while switching Leader/Follower roles. Pianists completed a total of 48 repetitions of each melody (4 conditions × 3 trials × 4 repetitions × 2 melodies = 96 repetitions in total). Participants received a nominal fee upon completion of the study.

### Data analysis

All timing analyses were based on MIDI tone onsets (1 ms temporal resolution). Consistent with other synchronization-continuation tasks that remove initial and final cycles of repetitive timing (Large et al. 2002; Goebl and Palmer 2009), the timing analyses were based on the middle two repetitions of each four-repetition trial, yielding a total of 64 keystrokes. The single whole note and half note in Piece 2 were interpolated at the quarter note level for purpose of comparison with Piece 1, resulting in 64 quarter note onsets per trial. Occasional pitch errors were identified by computer comparison of performances with the pitch content of a notated score (overall error rate <1 %). Repetitions in which pitch error additions or deletions occurred were excluded from analysis (5 % of repetitions for Piece 1; 7 % of repetitions for Piece 2). Three duet pairs did not follow the instruction to repeat the melody without pausing; instead, they paused between each repetition, resulting in final IOIs at the end of each repetition that were greater than three standard deviations above the mean. Their outlier IOIs were replaced with each subject’s mean IOI for the relevant serial position and condition (3 % of total IOIs for Piece 1 and 10 % of total IOIs for Piece 2). Outliers in measures of temporal coordination from duet performances, defined as values greater than three standard deviations from the mean, were excluded from further analyses.

Duet partners’ tone onset asynchronies, computed as Leader’s keystroke onsets − Followers’ keystroke onsets (ms), measured the phase synchrony between partners’ tone onsets. Negative values indicated that Leaders’ tone onsets preceded Followers’ tone onsets. Analyses of IOI patterns measured the period relationships between partners’ performances. Mean IOI values across Solo performances provided a measure of similarity of partners’ performance rates, critical for the hypothesis that difference in partners’ Solo performance rates correspond to successful synchronization during duet performance. Correlations between the IOI patterns of each partner provided a measure of period adaptation between partners during duet performance. We compared Leader and Follower IOI series at lags of +1 IOI to examine how much the Follower adapted to the previous IOI from the Leader and −1 IOI to measure how much the Leader adapted to the previous IOI from the Follower (Goebl and Palmer 2009; Loehr and Palmer 2011; Palmer 2013).

## Results

### Spontaneous rates in Solo performance

**Fig. 2:**
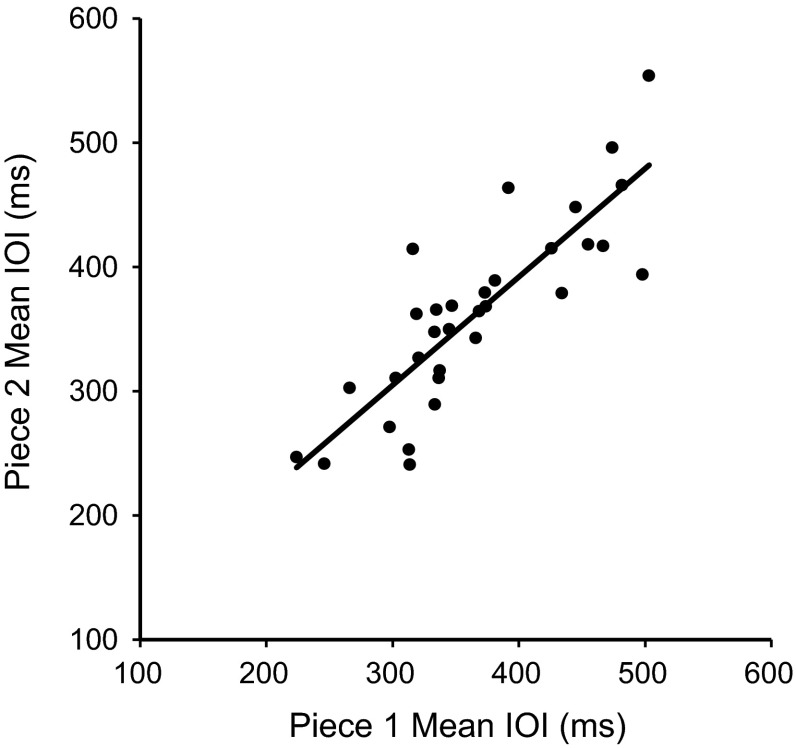
Mean spontaneous rates in Solo performance (quarter note duration, in ms) by stimulus melody for each performer

We first assessed the stability of pianists’ Solo performance rates across musical pieces. Figure 2 shows mean Solo performance rates, computed as the mean quarter note inter-onset interval, by performer for each musical piece; the mean rates correlated significantly, *r*(30) = .84, *p* < .01. There was no significant effect of trial on mean Solo rates, *F*(2, 60) = .76 *p* = .47. The same analysis performed on rank orderings of preferred rate also correlated significantly across musical sequences, *r*(30) = .87, *p* < .01. Consistency of absolute and rank ordered rates across musical pieces verifies that individual differences in Solo rate are robust across different performances and musical pieces and suggests that Solo rate is a stable measure.

### Duet synchronization measures

**Fig. 3:**
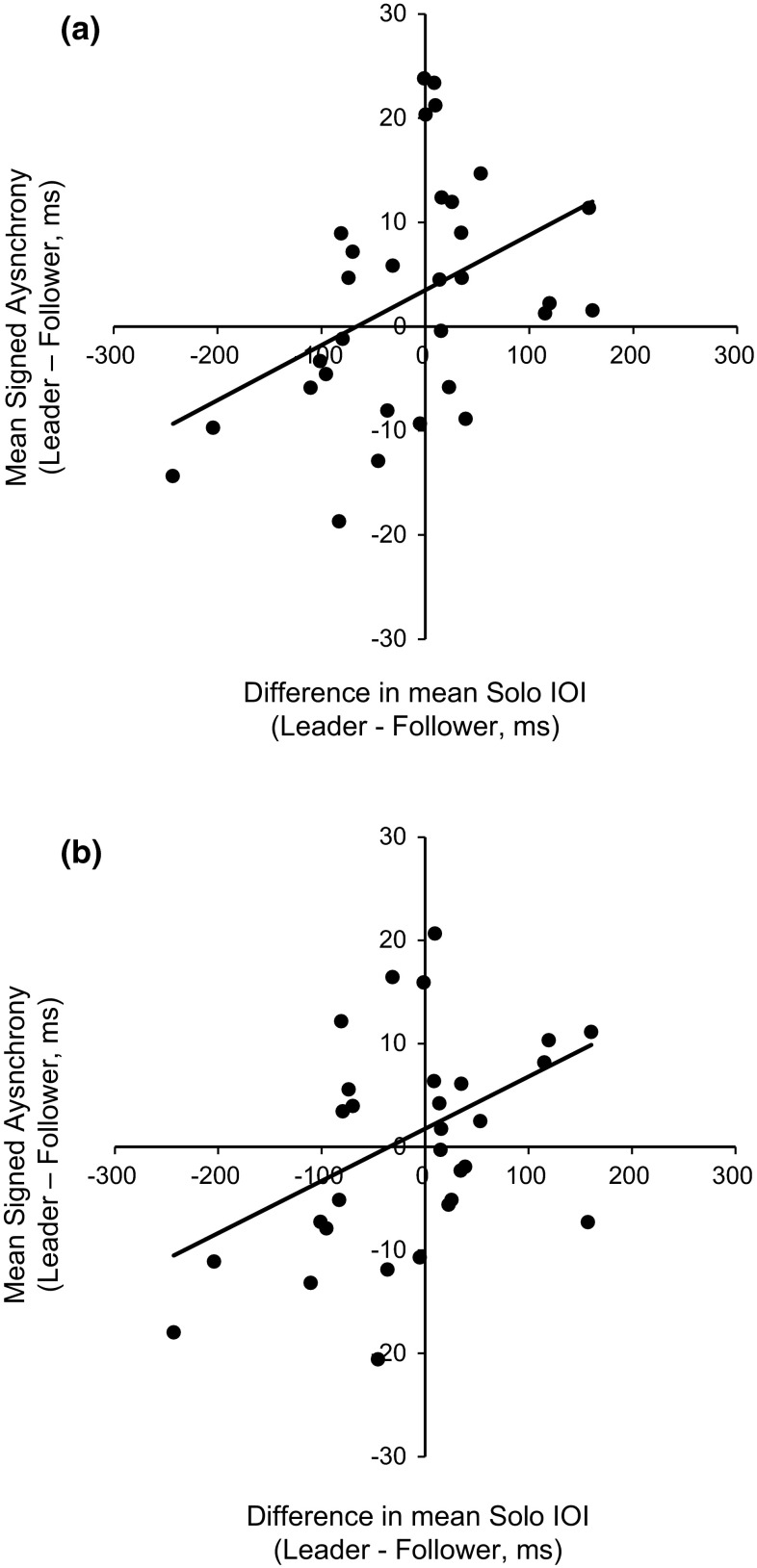
Mean signed asynchrony in Duet performances (Leader onset–Follower onset, in ms) by difference in mean Solo rates (Leader − Follower, in ms) for Unison–Full (**a**) and Unison–Other (**b**) conditions

We next assessed the relationship between partners’ Solo spontaneous rates and duet synchronization. Differences in duet partners’ mean Solo rates were correlated with their mean signed asynchrony during Unison duet performances (the condition most similar to the Solo performance task). As shown in Fig. 3, the difference in Solo spontaneous rate (Leader − Follower) correlated significantly with the pair’s duet asynchrony (Leader − Follower) during both Unison-Full performances, *r*(29) = .42, *p* < .05, and Unison-Other performances, *r*(29) = .39, *p* < .05; the larger the difference in preferred Solo rates, the larger the asynchrony in joint performance. The positive correlations indicate that the duet partner with the faster Solo rate tended to precede their partner in duet performances. The asynchronies in duet performances did not correlate with individual Leaders’ Solo rates, or with individual Followers’ Solo rates; only the difference between partners corresponded to their joint synchronization measures. The same analysis applied to Rounds performance did not yield significant correlations (Round–Full: *r*(29) = −.29, *p* = .12, Round–Other: *r*(29) = −.27, *p* = .14).

**Fig. 4:**
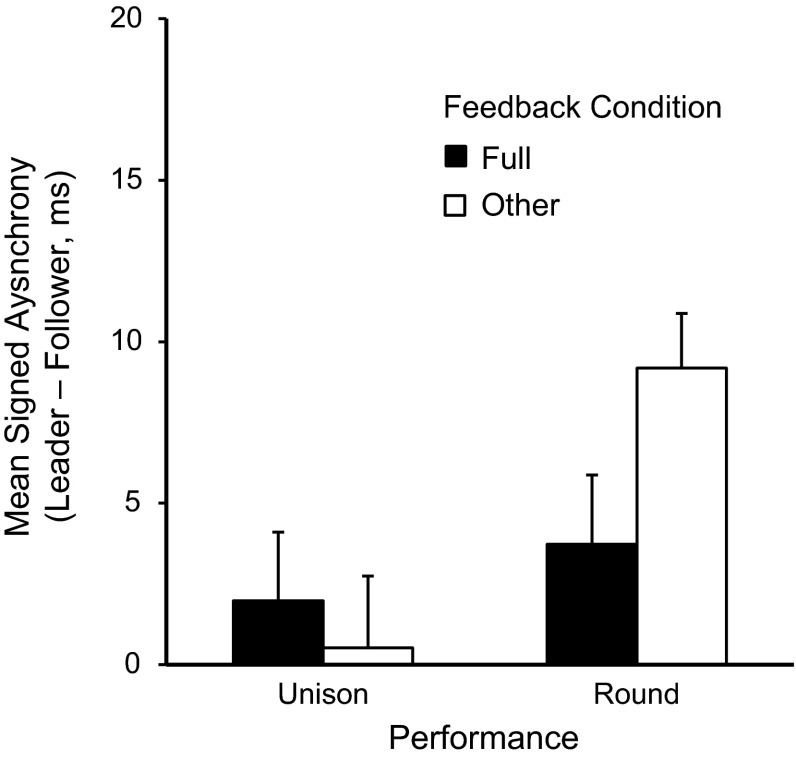
Mean signed asynchrony (Leader onset minus Follower onset, in ms) by Performance and Feedback conditions

Figure 4 shows the mean asynchrony values in duet performances by Performance and Feedback conditions. The mean signed asynchronies revealed a main effect of Performance, *F*(1,15) = 8.64, *p* = .01, with larger asynchronies for Round than Unison performances. There was also a main effect of Feedback, *F*(1,15) = 9.54, *p* < .01, and a significant interaction between Performance and Feedback, as shown in Fig. 4, *F*(1,15) = 8.12, *p* = .01. Mean asynchronies were significantly lower for Unison performances than for Round performances within the Other feedback conditions (Bonferroni-adjusted post hoc comparisons, *t*(15) = −4.60, *p* < .001) but did not differ between Unison and Round performances within the Full feedback conditions (*p* = .41).

### Inter-onset interval timing patterns

We next assessed patterns of period adaptation during duet performance with lag correlations, which measure the similarity of partners’ IOI time series at different temporal lags. Lags were defined relative to the Leader’s IOIs (the time series of the Follower was shifted relative to that of the Leader, while holding the time series length *L* constant): at lag0, the Leader’s IOI *N* was correlated with the Follower’s IOI *N,* across IOIs 33–96 (the middle two cycles). At lag +1 the Leader’s IOI *N* (33–96) was aligned with the Follower’s IOI *N* + 1 (34–97), and at lag −1, the Leader’s IOI *N* was aligned with the Follower’s IOI *N* *−* 1(32–95). Positive correlations at lag0 indicate similarity of simultaneous timing. Positive correlation coefficients at lag +1 indicate that the Follower is adapting to the Leader, whereas positive values at lag −1 indicate that the Leader is adapting to the Follower. Positive correlations at both +1 and −1 lags indicate bidirectional adaptation between performers.

**Fig. 5:**
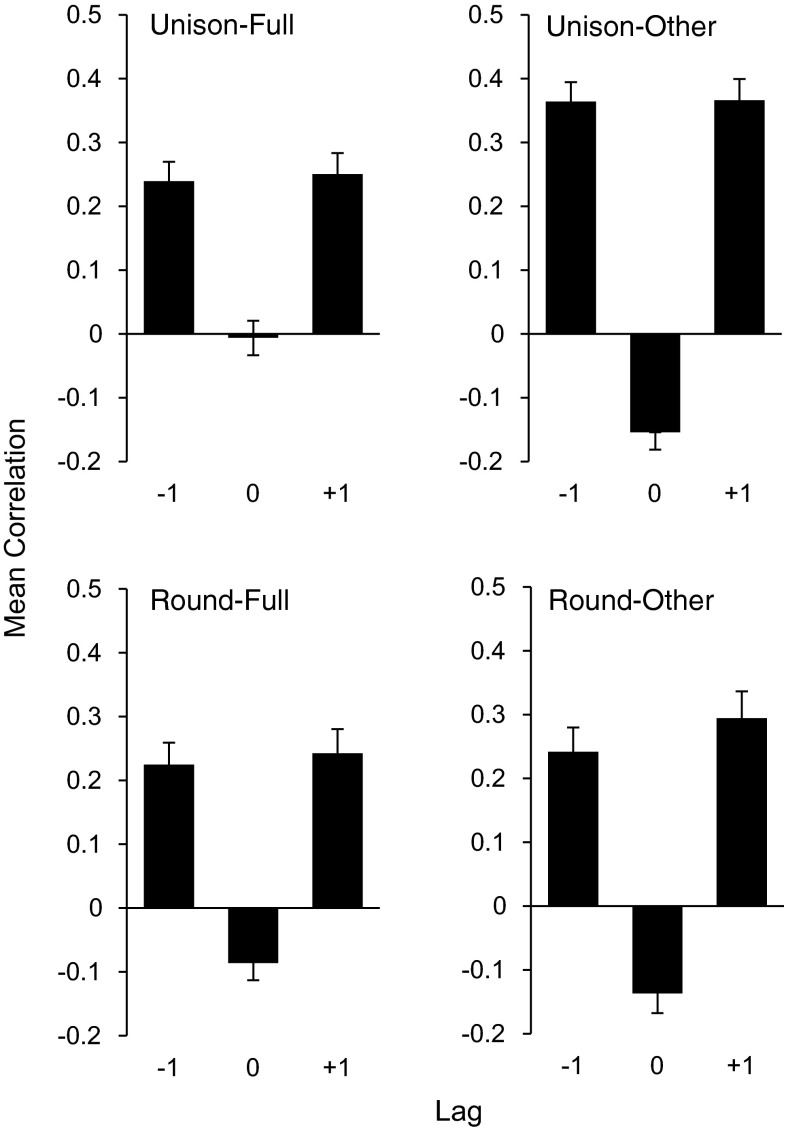
Mean correlations (lags −1, 0, +1) of partners’ Duet IOI patterns by Performance and Feedback conditions. *Rows* correspond to performance conditions Unison (*top*) and Round (*bottom*). *Columns* correspond to feedback conditions Full (*left*) and Other (*right*)

Figure 5 shows the mean correlation coefficients at each lag for Performance and Feedback conditions. An ANOVA on Performance (Unison vs. Round), Feedback (Full vs. Other) and Lag (−1, 0, 1) revealed a main effect of Lag on correlation coefficients, *F(*2,30) = 39.96, *p* < .001; correlations at lag0 indicated values close to zero, whereas positive correlations at both lags −1 and +1 indicated adaptation by both performers. Comparison of the correlation coefficients with critical Pearson values revealed significant values at lags +1 and  −1, but not at lag0: both duet partners showed significant temporal adaptation at lags +1 and −1, but did not show similar IOI patterns in real time, consistent with previous studies of piano duet performance (Goebl and Palmer 2009; Loehr and Palmer 2011). There were no significant differences in strength of lag +1 and −1 correlations (*p* > .1), suggesting that Leaders and Followers adapted to one another equally across both lags. Additionally, there were no significant differences in mean IOI across duet conditions (=395 ms, SE = 9.4 ms), confirming that duet performances maintained a consistent global tempo: Pianists were generally faster than the prescribed 500-ms metronome IOI, consistent with previous continuation tasks in piano performance (Pfordresher 2003). To further investigate patterns of period adaptation, subsequent analyses were conducted on lags +1 and −1.

**Fig. 6:**
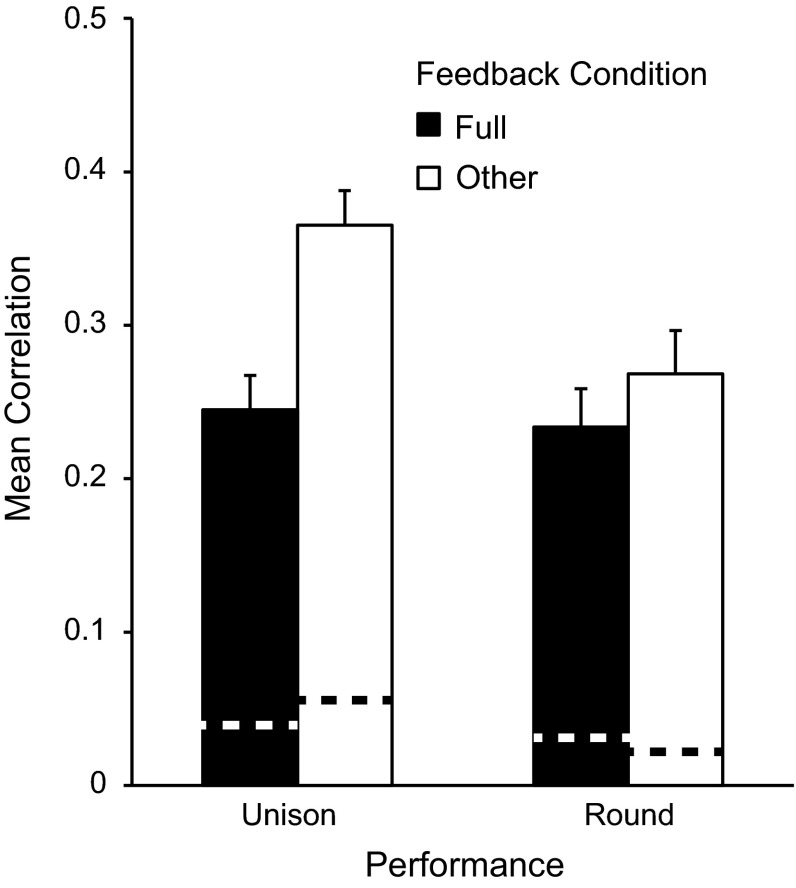
Mean correlations (averaged across lags +1 and −1) of partners’ Duet IOI patterns by Performance and Feedback conditions. *Dashed lines* represent chance estimates from jackknife procedure (see text)

Figure 6 shows the mean correlation coefficients by Performance and Feedback condition, for lags +1 and −1. There was a main effect of Performance on lag correlations, *F*(1,15) = 8.59, *p* = .01: Higher correlations were observed in Unison performances than in Round performances. Additionally, there was a main effect of Feedback, *F*(1,15) = 41.89, *p* < .001, and a significant interaction between Performance and Feedback, *F*(1,15) = 10.34, *p* < .01. Correlation coefficients were greater for Other feedback conditions than for Full feedback in Unison conditions, *t*(15) = 4.4, *p* < .001, Bonferroni correction), but not in Round performances (all *p’*s > .05). Thus, temporal adaptation was increased when performers did not have access to their own feedback, and when the pitch contents of auditory feedback from the partner matched the outcomes of one’s own performance.

Finally, we assessed whether pianists’ temporal adaptation was specific to each duet pair. The observed lag correlations for duet performances were compared with a chance estimate based on correlations between random pairings of pianists who did not perform together (jackknife procedure). The Leader’s IOI pattern on each duet trial was correlated with that of all possible Follower trials (except the original partner) within the same condition, resulting in 2,138 random pairings for all conditions. The upper confidence intervals, a conservative estimate, were computed for the mean correlations within each condition. Paired-sample *t* tests (Bonferroni-corrected) between correlation coefficients for observed values and the upper confidence interval values from random pairings indicated significantly larger observed values at lags +1 and −1 for each Performance × Feedback condition (all *ps* < .05). These findings suggest that partner-specific adaptation occurred in each of the duet performance conditions, at a rate substantially higher than chance.

## Discussion

Temporal coordination of tones in joint music performance is challenging: Partners must maintain temporal regularity while simultaneously adjusting in real time to auditory feedback from their partner. Two important sources of temporal variability affected musicians’ ability to synchronize with their partner: individual differences in partners’ spontaneous (uncued) rates of performance, and auditory feedback arising from self- and partner-generated actions. In a naturalistic task, duet pianists performed melodies independent of a partner (Solo), at the same time as a partner (Unison), or at a temporal offset (Round). The alignment of self- and partner-produced auditory information was manipulated across Unison and Round conditions while the auditory feedback generated from one’s own performance was held constant across these conditions. Performers heard either feedback from themselves and their partner (Full), or they heard only feedback from their partner (Other). Measures of spontaneous performance rate in Solo performances and synchronization of tone onsets in duet performances provided evidence that individual differences in endogenous rhythms, as well as differences in self- and partner-generated auditory feedback, influence temporal coordination of this joint sensorimotor behavior. Each finding is discussed in turn.

First, temporal coordination between duet partners increased as differences in their spontaneous Solo performance rates decreased. Notably, synchronization between duet partners did not correlate with either performer’s spontaneous rate, but rather with the difference between partners’ spontaneous rates, similar to previous findings in joint musical tasks (Loehr and Palmer 2011). These observed links between spontaneous rates and increased coordination in duet performances are consistent with entrainment accounts that predict facilitated coordination when endogenous rhythms match the temporal properties of external auditory signals (Large and Jones 1999; Large and Palmer 2002; Nozaradan et al. 2013), and with findings that musicians synchronize better with recordings of their own performances than with other’s performances (Keller et al. 2007). Similarity of the partners’ spontaneous performance timing corresponded to joint synchronization only when performers produced the same melody together (Unison) and not when they produced different pitch patterns (Rounds). Endogenous oscillators may play a greater role in predicting synchronization during tasks in which partners produce the same auditory feedback and/or the same actions; the current study does not distinguish these alternatives. Whether partners’ similar endogenous rhythms are related to biomechanical or anatomical differences across effectors is another question for future research.

Second, temporal coordination in duet performance increased as the similarity between self- and partner-generated auditory feedback increased. Unison performances (in which self- and other-based feedback were identical) yielded better synchronization than Rounds, in which pianists heard their partner produce a serially shifted copy of their own sequence. Round performances were associated with both lower correlations and larger asynchronies, relative to Unison performances. These findings are consistent with the hypothesis that similarity-based interference arises from conflict between intended and heard events (Palmer and Pfordresher 2003; Pfordresher 2005; Pfordresher and Palmer 2006). Notably, asynchronies during Round performances indicated that Followers’ keystroke onsets preceded Leaders’ keystroke onsets: This directionality is opposite to that of the serially shifted feedback. Although the current analyses cannot distinguish whether Followers were attempting to catch up to Leaders, or whether Leaders were lagging behind Followers, the directionality suggests compensatory timing behavior: followers rushed to “catch up” with the future, or Leaders lagged because they heard feedback from the past. This compensatory behavior is consistent with previous feedback manipulations in Solo music performance and in speech (Finney 1997; Dell 1986; Houde and Jordan 1998), suggesting that compensatory mechanisms in individual sensorimotor behaviors may also come into play during coordination of joint sensorimotor behaviors. These findings also suggest that relationships between the content of self- and partner-generated feedback play an important role in temporal coordination of joint behaviors.

Finally, temporal coordination of tone onsets during duet performance decreased when self-feedback was removed from Rounds performances. In contrast, removal of self-feedback did not disrupt the synchronization of Unison performances. Pianists’ adaptation to their partner’s timing, indicated by higher lag correlations, increased in this condition when self-feedback was removed. These findings suggest that performers may not require their own auditory feedback to successfully coordinate the timing of actions when auditory feedback from a partner matches self-feedback (Unison), consistent with previous work showing that removal of self-feedback does not disrupt production of well-learned sequences during Solo performance (Finney and Palmer 2003) or during joint performance (Goebl and Palmer 2009). In contrast, performers may rely more on self-feedback when feedback from a partner creates similarity-based interference with their own performance.

In many real-world contexts, musicians must perform with partners whose musical parts and performance timing differ from their own. The overall level of temporal coordination observed in this study of skilled musicians was high: Similar to previous findings of skilled performance, pianists’ tone onsets were on average within 30 ms of each other (Goebl and Palmer 2009; Loehr and Palmer 2011), and they adapted to their partner even in the more challenging Round performances. Novice performers may show greater disturbances during removal of auditory feedback or interference from one’s partner’s feedback. Less skilled performers tend to display less flexibility and temporal adaptation to changing auditory signals (Drake and Palmer 2000; Repp 2010), and may be more susceptible to interference from a partner’s auditory feedback, compared with skilled musicians (Hansen et al. 2013). Thus, partner-specific timing differences and sensitivity to removal of auditory feedback may be more pronounced in novice performers. Because many joint sensorimotor behaviors require coordination between non-expert individuals who are not familiar with their partners, from working with new colleagues to playing amateur athletics, it is important to understand how different endogenous rhythms and different sensory feedback may constrain temporal coordination during joint sensorimotor tasks.
